# Neolignans isolated from *Saururus cernuus* L. (Saururaceae) exhibit efficacy against *Schistosoma mansoni*

**DOI:** 10.1038/s41598-022-23110-2

**Published:** 2022-11-11

**Authors:** Juliana R. Brito, Polrat Wilairatana, Daniel B. Roquini, Beatriz C. Parra, Marina M. Gonçalves, Dalete Christine S. Souza, Edgard A. Ferreira, Maria C. Salvadori, Fernanda S. Teixeira, João Henrique G. Lago, Josué de Moraes

**Affiliations:** 1 Institute of Environmental, Chemical and Pharmaceutical Sciences, Federal University of São Paulo, Diadema, SP 09972-270 Brazil; 2 Department of Clinical Tropical Medicine, Faculty of Tropical Medicine, Mahidol University, Bangkok, 10400 Thailand; 3 Research Center on Neglected Diseases, Guarulhos University, Guarulhos, SP 07023-070 Brazil; 4 Center for Natural and Human Sciences, Federal University of ABC, Santo André, SP 09210-180 Brazil; 5 School of Engineering, Mackenzie Presbyterian University, São Paulo, SP 01302-907 Brazil; 6 Institute of Physics, University of São Paulo, São Paulo, SP 05508-090 Brazil

**Keywords:** Antiparasitic agents, Parasitology

## Abstract

Schistosomiasis, a parasitic disease caused by the blood fluke of the genus *Schistosoma*, affects over 230 million people, especially in developing countries. Despite the significant economic and public health consequences, only one drug is currently available for treatment of schistosomiasis, praziquantel. Thus, there is an urgent demand for new anthelmintic agents. Based on our continuous studies involving the chemical prospection of floristic biodiversity aiming to discover new bioactive compounds, this work reports the in vitro antiparasitic activity against *Schistosoma mansoni* adult worms of neolignans *threo*-austrobailignan-6 and verrucosin, both isolated from *Saururus cernuus* L. (Saururaceae). These neolignans showed a significant in vitro schistosomicidal activity, with EC_50_ values of 12.6–28.1 µM. Further analysis revealed a pronounced reduction in the number of *S. mansoni* eggs. Scanning electron microscopy analysis revealed morphological alterations when schistosomes were exposed to either *threo*-austrobailignan-6 or verrucosin. These relevant antischistosomal properties were accompanied by low cytotoxicity potential against the animal (Vero) and human (HaCaT) cell lines, resulting in a high selectivity index. Considering the promising chemical and biological properties of *threo*-austrobailignan-6 and verrucosin, this research should be of interest to those in the area of neglected diseases and in particular antischistosomal drug discovery.

## Introduction

Schistosomiasis, a poverty-associated parasitic disease caused by blood fluke of the genus *Schistosoma,* is a debilitating disease with a tremendous global burden. Estimates show that over 230 million people are affected with schistosomiasis in 78 countries and approximately 10% of the world population is at risk for infection^1^. *Schistosoma mansoni*, one of the three major human species, occurs across much of Africa, the Middle East, the Caribbean, and South America. Morbidity due to schistosomiasis mansoni includes hepatosplenomegaly, liver fibrosis, and ascites; in severe cases, *S. mansoni* infection can be fatal^2^. Consequently, it is a disease of immense medical importance.

The control of schistosomiasis is mainly dependent on the use of praziquantel, the only readily commercially available drug^3,4^. The Word Health Organization (WHO) strategy for schistosomiasis control focuses on large-scale treatment (preventive chemotherapy) of affected populations, a strategy that might select for drug-resistant parasites. In addition, numerous persistent schistosomiasis hotspots remain^5,6^, and low cure rates have been reported^7,8^. Systematic reviews and meta-analyses show that praziquantel achieves a cure rate of approximately 75% for *S. mansoni* infections^9,10^, demonstrating the limitations of praziquantel. With the aim to eliminate human schistosomiasis as a public health problem by 2030, in their new road map for neglected tropical diseases 2021–2030, the WHO highlights the need for new therapeutic interventions^11^. Thus, the pressing need to develop new anthelmintic compounds has been emphasized and the drug discovery landscape has gained momentum over the past few years^12–14^.

*Saururus cernuus* L. (*S. cernuus,* Saururaceae) is a type of freshwater plant widely distributed in America, including Brazil^15^. In folk medicine, this plant has been used as an anti-inflammatory and as a sedative^15,16^. Phytochemically, *S. cernuus* produces mainly lignoids but the occurrence of other compounds such as alkaloids and terpenoids was also reported^17–19^. Studies describing antiprotozoal effects of some lignoids isolated from *S. cernuus* were previously described, including *threo,threo*- and *threo,erythro*-manassantin A with activity against amastigote forms of *Leishmania amazonensis*^20^ and *threo*-austrobailignan-5, *threo*-austrobailignan-6, and *threo*-dihydroguaiaretic acid with activity against trypomastigote and amastigote forms of *Trypanosoma cruzi*^21^. Furthermore, alterations in the plasma membrane permeability, in reactive oxygen species (ROS) levels, and mitochondrial membrane potential caused by these compounds in tested parasites were observed. More recently, the molecular dereplication and the evaluation of anti-*T. cruzi* activity of volatile oils from inflorescences, leaves, branches, and roots of *S. cernuus* was reported. As described, the predominance of mono and sesquiterpenes was observed, as well as phenylpropanoids as the main compounds. Interestingly, oils from leaves, branches, inflorescences, and roots displayed activity against trypomastigotes, with reduced toxicity against NCTC cells^22^. This selectivity makes these compounds attractive as antiparasitic agents.

As part of our continuous study aiming to discover new antischistosomal agents from plants of Brazilian biodiversity^23–25^, this study reports the isolation of two neolignans from leaves of *S. cernuus,* of which one is dibenzylbutane—threo-austrobailignan-6—and one tetrahydrofuran—verrucosin—and their evaluation of anthelmintic properties against *S. mansoni* was performed, since these compounds exhibited activity against other parasites such as *T. cruzi* and *L. infantum*.

## Results and discussion

### Chemical characterization of *threo*-austrobailignan-6 and verrucosin

**Figure 1:**
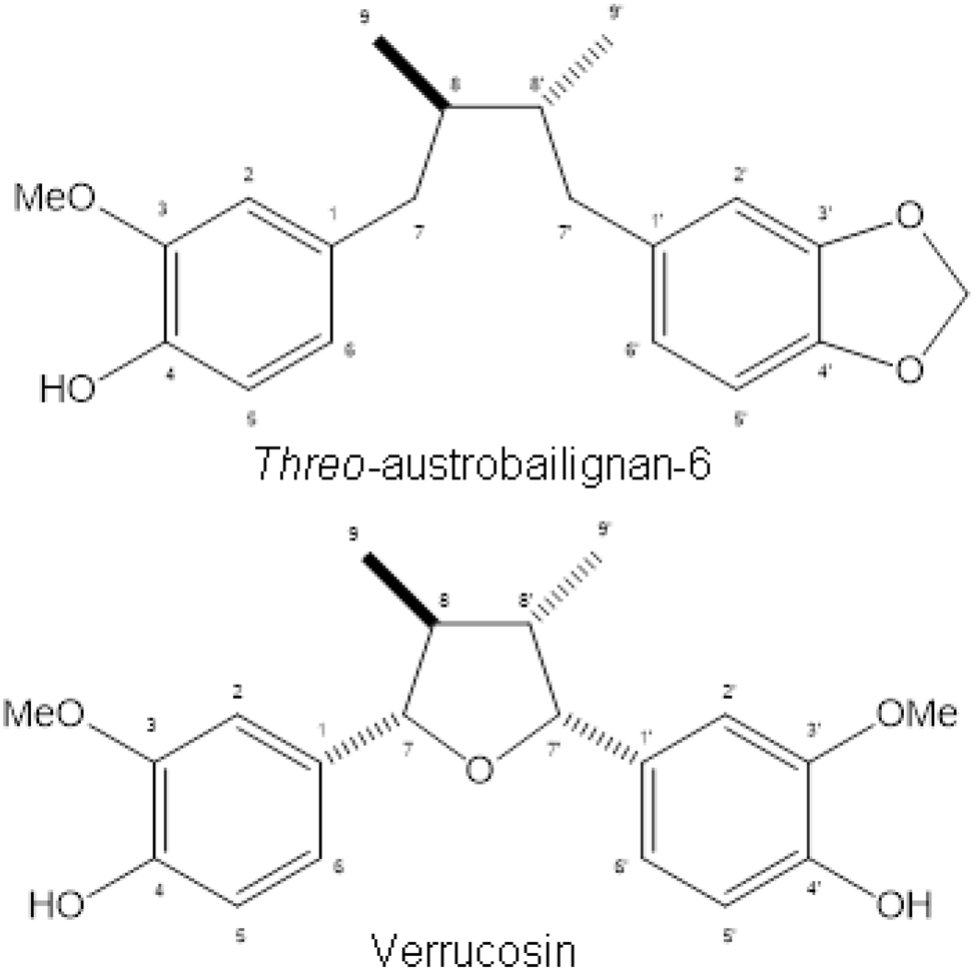
Chemical structures of neolignans isolated from *S. cernuus.*

NMR spectral data of *threo*-austrobailignan-6 were identical to those reported in the literature^26^. As observed, the ^1^H NMR spectrum indicated the presence of two 1,3,4-trissubtituted aromatic rings due the signals at δ 6.67 (d, *J* = 1.7 Hz, H-2), 6.70 (d, *J* = 7.8 Hz, H-5), 6.60 (d, *J* = 1.7 Hz, H-2′), 6.80 (d, *J* = 7.9 Hz, H-5′), and 6.57–6.53 (m, H-6 and H-6′). The presence of four double-doublets at δ 2.39 (*J* = 13.5 and 8.0 Hz), 2.57 (*J* = 13.5 and 6.5 Hz), 2.33 (*J* = 13.5 and 8.0 Hz), and 2.56 (*J* = 13.5 and 6.5 Hz), assigned to H-7a, H-7b, H-7′a and H-7′b, respectively, in association with two doublets at δ 0.86 (*J* = 5.9, H-9) and 0.81 (*J* = 6.6, H-9′), indicated the occurrence of a dibenzylbutane neolignan^21^. Two singlets at δ 5.93 (2H) and 3.84 (3H) indicated the presence of methylenedioxy and methoxy groups, respectively, in the aromatic rings. ^13^C NMR spectrum of shown signals referring to aromatic carbons from δ 107.9 to 147.3 (C-1 to C-6 and C-1′ to C-6′), two methylene carbons at δ 41.1 (C-7) and 41.0 (C-7′), two methine carbons at δ 37.9 (C-8) and 37.8 (C-8′), and two methyl groups at δ 13.9 (C-9) and 13.8 (C-9′). Furthermore, signals at δ 100.9 and 55.8 were assigned to methylenedioxy and methoxy groups, respectively. Finally, based on the chemical shifts of C-8/C-8′ and C-9/C-9′^27^, it was possible to infer that methyl groups at C-8 and C-8′ are positioned at *trans* configuration (Fig. 1).

NMR spectral data of verrucosin were similar to those reported in the literature^28^. Analysis of its ^1^H NMR spectrum, which showed a multiplet at δ 6.97—6.76 (H-2, H-5, H-6, H-2′, H-5′ and H-6′), two doublets of oxymethine hydrogens at δ 5.03 (*J* = 8.7, H-7) and 4.33 (*J* = 9.3, H-7′) and two doublets of methyl groups at δ 0.98 (*J* = 6.5 Hz, H-9) and 0.59 (*J* = 7.0 Hz, H-9′), indicated the occurrence of a tetrahydrofuran neolignan^29^. Additional singlets at δ 5.54 (4-OH), 5.49 (4′-OH), and 3.84 (3- and 3′-OMe) inferred the presence of two *ortho* methoxy-phenol moieties. ^13^C NMR spectrum showed signals referring to aromatic carbons from δ 109.4 to 146.5 (C-1 to C-6 and C-1′ to C-6′), two oxymethine carbons at δ 87.3 (C-7) and 83.1 (C-7′), two methine carbons at δ 47.8 (C-8) and 46.0 (C-8′), and two methyl groups at δ 14.5 (C-9) and 14.9 (C-9′). Furthermore, one intense signal at δ 55.9 was attributed to methoxyl groups at C-3 and C-3′. Based on the chemical shifts of C-7/C-7′, C-8/C-8´and C-9/C-9′^30^, it was possible to infer the stereochemistry *trans,trans,cis* to the substituents in the tetrahydrofuran groups (Fig. 1).

Finally, molecular formulas of isolated compounds were confirmed as C_20_H_24_O_4_ and C_20_H_24_O_5_ based on ESI-HRMS spectral data—*threo*-austrobailignan-6 shown [M—H]^−^ ion peak at *m/z* 327.1594 whereas verrucosin exhibited [M + H]^+^ and [M + Na]^+^ ion peaks at *m/z* 345.1798 and 367.1518, respectively.

### *Threo*-austrobailignan-6 and verrucosin affected the viability on adult *S. mansoni*

**Figure 2:**
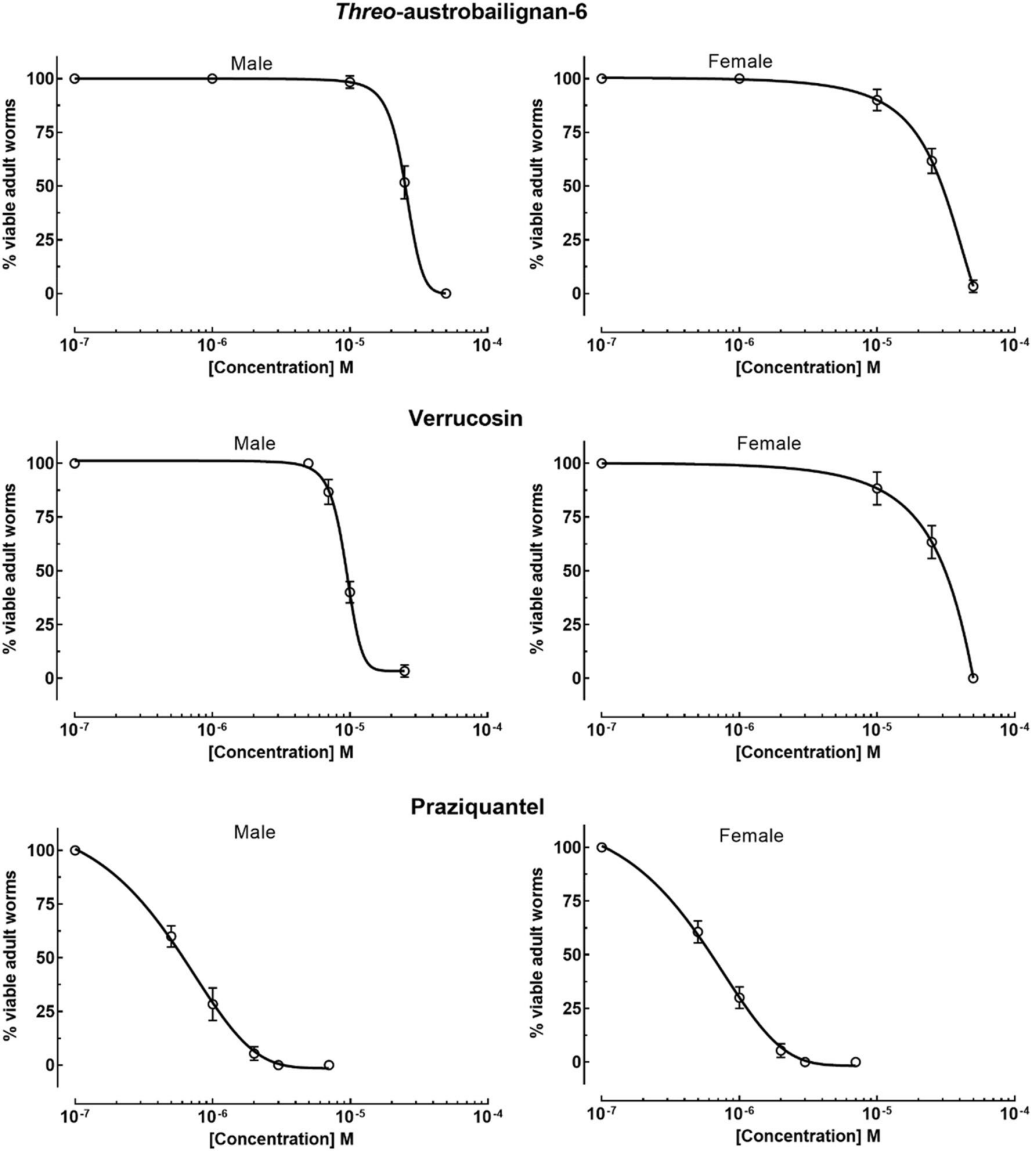
Concentration–response curves for threo-austrobailignan-6, verrucosin, and praziquantel. Viability of ex vivo adult *S. mansoni* worms (male and female) was recorded within 72 h. Data are presented as the mean ± SD from a minimum of three experiments (*n* = 3). EC_50_ values are shown in Table 1.

As reviewed elsewhere^31^, pharmacological studies demonstrated that plants of the genus *Saururus* have several compounds with biological activities, including anti-inflammatory and antitumor properties as well as antiprotozoal activities^20,21^. However, to the best of our knowledge, the effect of extracts or compounds from *Saururus* against human parasitic worms has not been described so far. In this study, to determine the antischistosomal potential of *threo*-austrobailignan-6 and verrucosin, *S. mansoni* worm pairs (male and female) were exposed to different concentrations for their effective concentration 50% (EC_50_) determination (Fig. 2). The known antiparasitic drug praziquantel was used as a reference. There were no modifications in the viability of the adult schistosomes belonging to the negative control groups for 72 h. As shown in Table 1, both verrucosin and *threo*-austrobailignan-6 presented schistosomicidal activity with EC_50_ values below 30 μM. Comparatively, verrucosin exhibited superior antiparasitic efficacy than *threo*-austrobailignan-6, with EC_50_ values ranging from 12.6 to 18.7 μM and 22.9 to 28.1 μM, respectively. The reference drug praziquantel was confirmed to be highly active, with an EC_50_ below 0.93 μM.

**Table 1:** In vitro activities of *threo*-austrobailignan-6, verrucosin, and praziquantel against *S. mansoni* adult worms and cytotoxicity.

Compounds	*S. mansoni* EC_50_ (μM)		Monkey cells		Human cells	
			CC_50_ (μM)	SI	CC_50_ (μM)	SI
	Male	Female				
*Threo*-austrobailignan-6	22.9 [18.2–28.6]*	28.1 [23.6–34.7]*	> 500	> 17.8	> 500	> 17.8
Verrucosin	12.6 [10.8–18.3]*	18.7 [15.2–22.1]*	> 500	> 26.8	> 500	> 26.8
Praziquantel	0.7 [0.6–0.8]	0.8 [0.7–1.0]	> 500	> 714	> 500	> 714

EC_50_, Effective concentration 50% against adult schistosomes; CC_50_, Cytotoxic concentration 50% against monkey (Vero) and human (HaCaT) cells; SI, Selectivity Index; *95% Confidence interval. The parasites were exposed for 72 h to the tested compounds to calculate the EC_50_. Values are calculated from three experiments, and each experiment was performed with three replicates. ND, not determined.

Based on the results obtained, tetrahydrofuran lignans, mainly verrucosin, have a potent schistosomicidal activity, and these compounds are more effective against schistosomes than other plant-derived products. For example, the following in vitro effective concentrations against *S. mansoni* adult worms were observed in the literature: 30 µM with neolignan licarin A^25^, 52 µM with monoterpene 3,7-dimethylloctanol^32^, 56.8 µM with monoterpene carvacrol^33^, 42.16 µM with carvacryl acetate, a derivative of carvacrol^34^, 85 µM with sesquiterpene nerolidol^35^, 50 µM with sesquiterpene cnicin^36^, 26.1 µM with diterpene *ent*-kaur-16-en-19-oic acid^24^, 50 µM with licochalcone A^37^, 50 µM 2-oxopopulifolic acid methyl ester, and 2-oxopopulifolic acid^38^, and 25 µM with chalcone cardamonin^39^.

**Figure 3:**
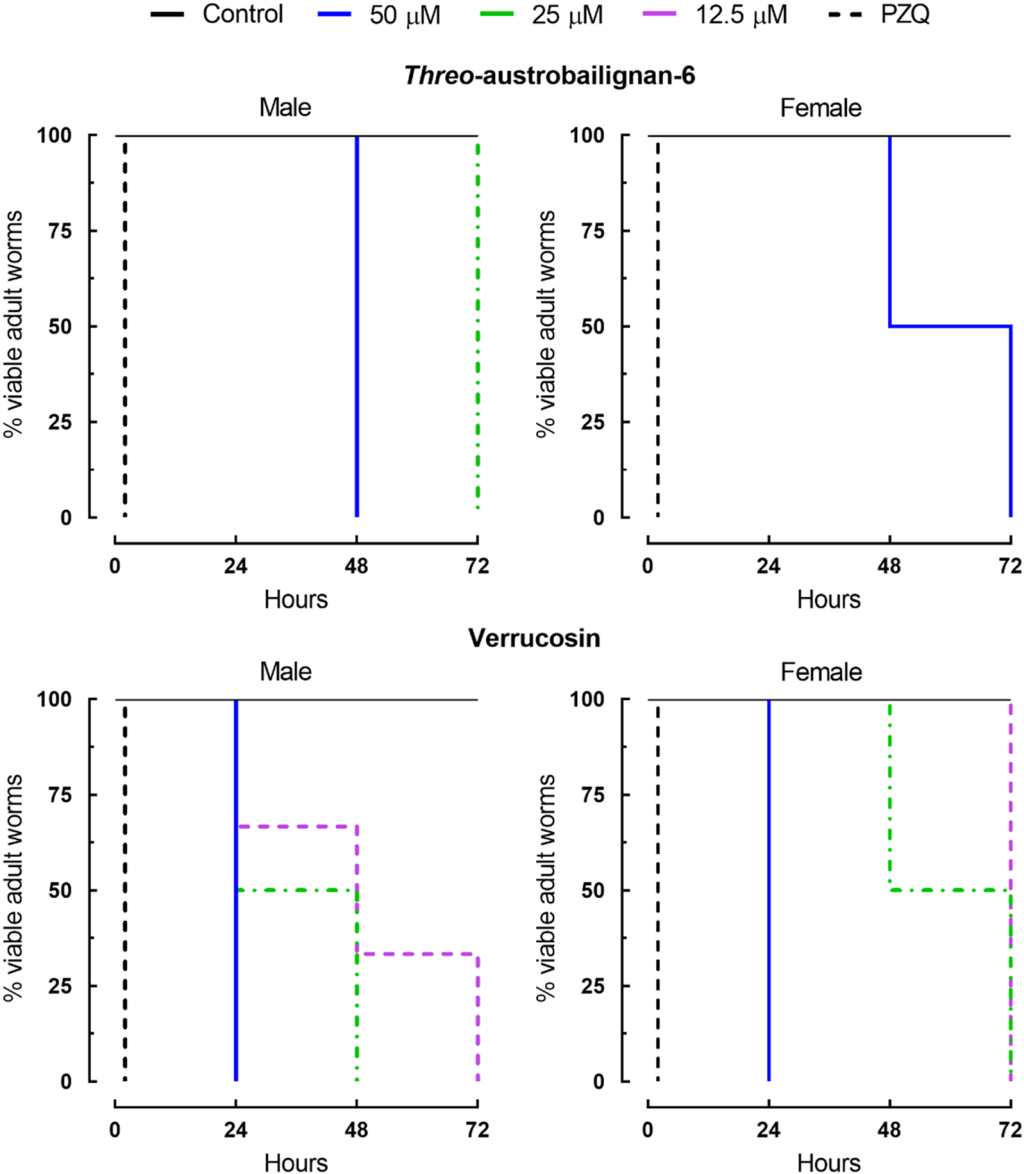
Viability of ex vivo adult *S. mansoni* worms following exposure to neolignans. Adult worm pairs were obtained from mice by perfusion 42 days after infection. Parasites were monitored for up to 72 h, and results are expressed as the percent mortality recorded by Kaplan–Meier survival curves. Mean values of viability were derived from a minimum of three experiments (*n* = 3). Control: drug-free medium. PZQ: praziquantel at 2 µM.

Assays regarding the survival times of adult worm pairs were also performed with tetrahydrofuran lignans, to understand the kinetics and mode of action of these substances. As shown in Fig. 3, these natural compounds induced mortality in a time- and concentration-dependent manner. Control parasites remained viable over the entire observation period, whereas praziquantel caused mortality of all worms immediately. The antischistosomal assay also revealed that verrucosin and *threo*-austrobailignan-6 were slightly more active against male *S. mansoni*. For example, verrucosin at 25 μM was lethal to 100% of male parasites after 48 h, whereas the death of all female worms was recorded within 72 h. When adult worm pairs were exposed to *threo*-austrobailignan-6 at a concentration of 25 μM for 72 h, 100% mortality was observed only for male worms. Similar to the anthelmintic properties of two tested neolignans, several studies have shown that male schistosomes are often more susceptible than female helminths^40–42^, and these results point to a sex-specific difference of tested compounds with the targets, but the mechanism of action remains to be elucidated. It should be noted that some compounds showed higher selectivity to female helminths, such as diterpene phytol^43^, or are equally active against both parasite sexes such as the prenylated benzophenone garcinielliptone FC^44^. It is known that male schistosomes had a higher sensitivity to praziquantel than female parasites^45^, corroborating the results obtained in this study. Multiple mechanisms are likely to be responsible for the difference in sensitivity between males and females exposed to verrucosin and *threo*-austrobailignan-6. Some of these may be due, in part, to the greater exposure of male worms to the surrounding environment. Indeed, because the *S. mansoni* female resides in the gynecophore canal, it may be less susceptible to attacks by anthelmintic molecules.

### *Threo*-austrobailignan-6 and verrucosin reduced *S. mansoni* egg production

**Figure 4:**
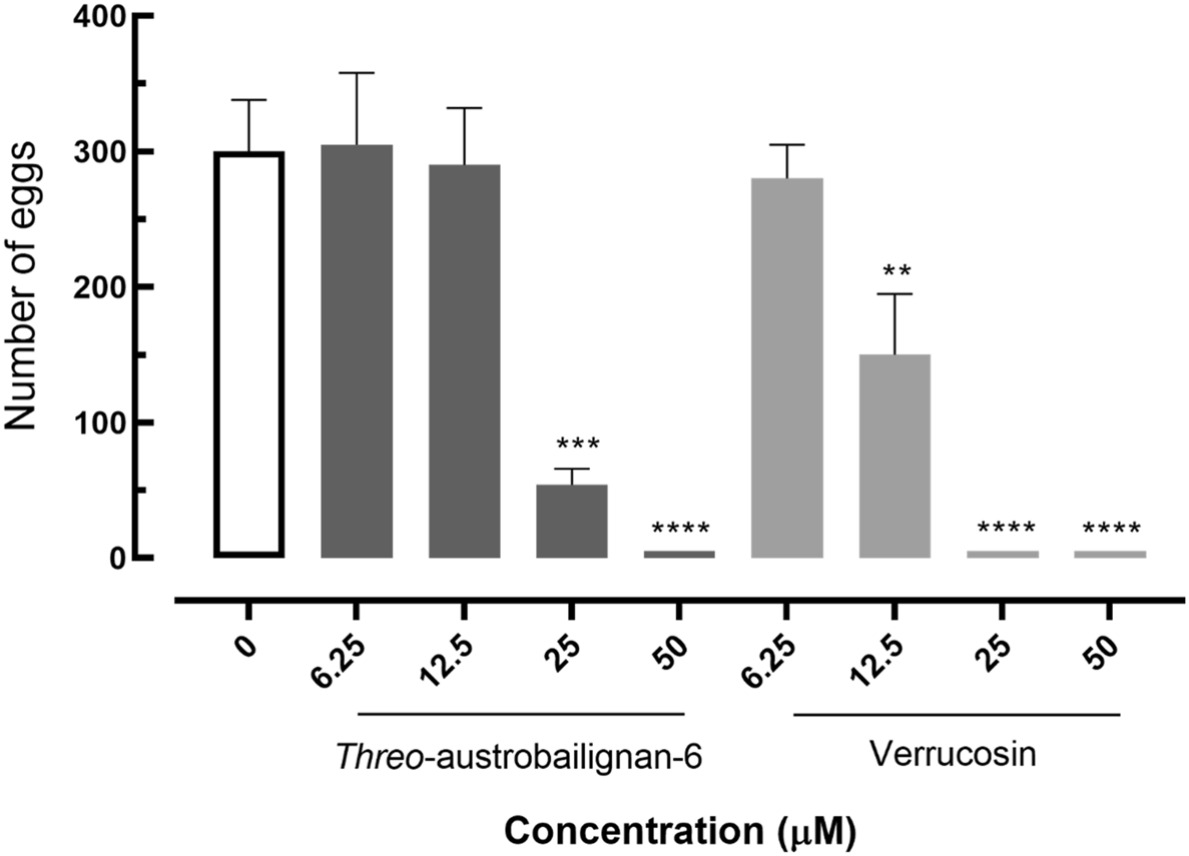
Eggs released by paired adult *S. mansoni* exposed to neolignans. Control: drug-free medium. Data are presented as the mean ± SD from three independent experiments (*n* = 3).

The ability of tested tetrahydrofuran lignans to affect *S. mansoni* egg production was measured and the number of eggs within 72 h is shown in Fig. 4. All schistosomes of the negative control group showed normal viability and they remained paired and active throughout the treatment. Incubation of adult helminth pairs with either *threo*-austrobailignan-6 or verrucosin at a concentration of 50 μM kept the male and female parasites separated, which prevented the mating process, and a complete lack of oviposition was observed. At a concentration of 25 μM the parasites remained coupled, but the total number of egg worms was significantly reduced when compared to control worms (*P* < 0.0001 and *P* < 0.001 for verrucosin or *threo*-austrobailignan-6, respectively). A significant reduction in oviposition was also recorded with verrucosin at 12.5 μM (*P* < 0.01).

Considering that egg production is an essential mechanism for both transmission and pathogenesis, the negative impact on schistosomes egg-laying is of particular interest for molecules with anthelmintic activities. Indeed, the effects of antischistosomal agents on egg output during in vitro incubation have been previously reported in several studies^46–49^. Similar to these studies, *threo*-austrobailignan-6 and verrucosin, which also had a schistosomicidal effect, caused a reduction in *S. mansoni* egg production. The interference in oviposition may be associated with the separation of parasite couples or by changes in the reproductive system of *S. mansoni*^42,49^.

### *Threo*-austrobailignan-6 and verrucosin caused morphological alterations to the schistosome tegument

**Figure 5:**
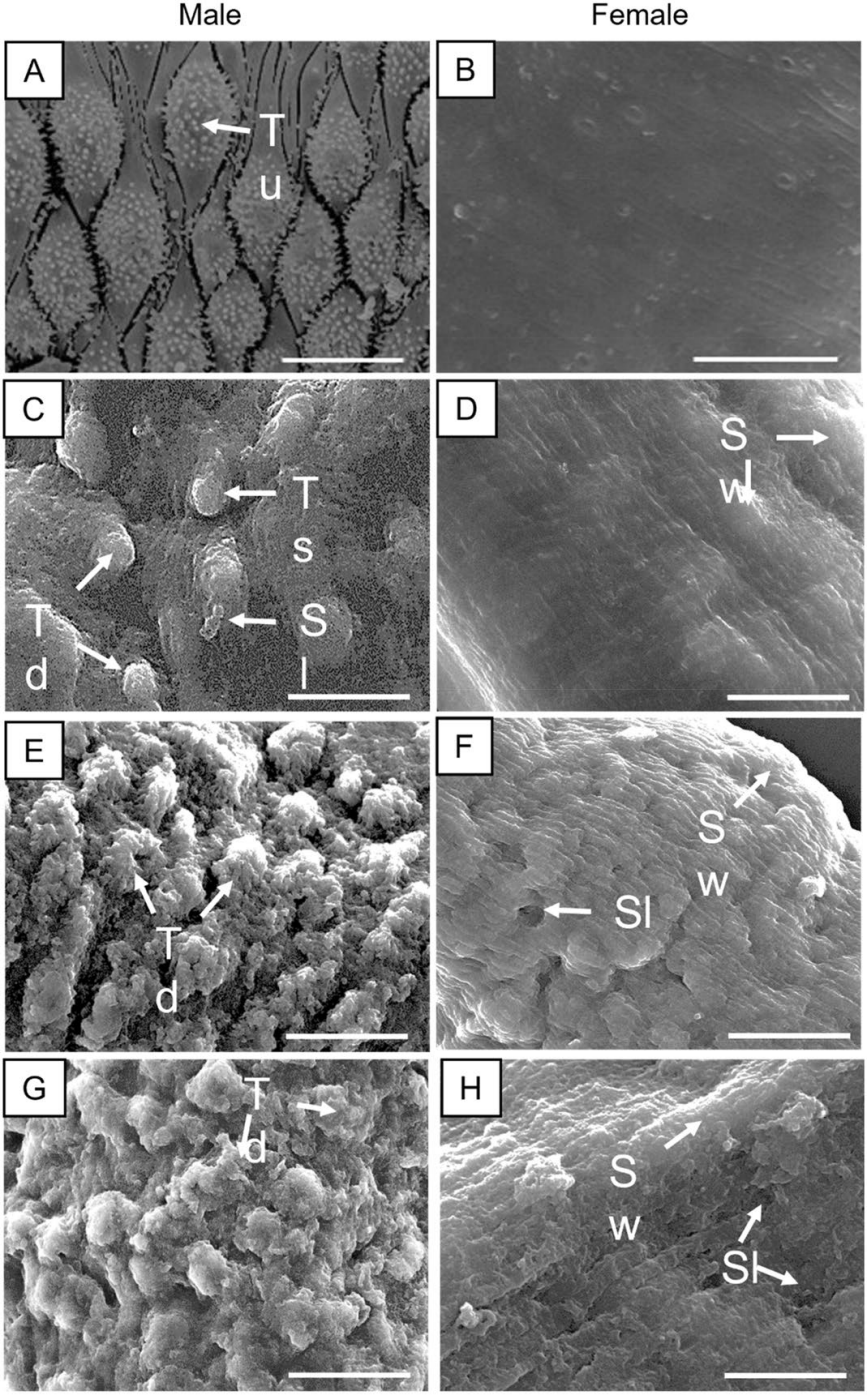
Scanning electron microscopy investigation of adult *S. mansoni* following incubation with neolignans. Control male worms showing intact tubercles (Tu) and spines on the surface (arrow) (**A**) and control female worms (**B**). Schistosomes were exposed to *threo*-austrobailignan-6 at 50 µM (**C** and **D**) or verrucosin at 25 µM (**E** and **F**) and 50 µM (**G** and **H**). Male (**A**, **C**, **E**, and **G**) and female (**B**, **D**, **F**, and **H**) helminths. The dorsal tegumental surface shows tubercle shortening (Ts), swelling (Sw), sloughing (Sl), and tubercle disintegration (Td). Images were captured using a JEOL SM 6460LV scanning electron microscope. Scale-bars: 10 µm.

Given the importance of the schistosomes’ tegument as a target for an anthelmintic agent^50^, we used scanning electron microscopy to examine the surface of *S. mansoni* adult parasite exposed to *threo*-austrobailignan-6 or verrucosin. The two neolignans tested at lethal concentrations caused morphological changes in the tegumental surfaces of male and female schistosomes. As shown in Fig. 5, adult parasites belonging to the control group (drug-free medium) showed an intact surface structure and topography (Fig. 5A,B). In contrast, helminths exposure to *threo*-austrobailignan-6 at 50 µM exhibited severe tegumental alterations such as swelling, sloughing, and shortening of the tubercles (Fig. 5C,D). Schistosomes incubated with verrucosin at 25 µM (Fig. 5E,F) and 50 µM (Fig. 5G,H) displayed substantial tegumental disruption throughout the whole body, with the tubercles losing their natural shape, and the spicules were markedly affected when compared to those of controls.

Morphological alterations induced by several antischistosomal natural compounds on the tegument of *S. mansoni* have also been described. For example, a disintegration of the schistosomes’ surface was also observed after exposure to epiisopilosine^48,51^, piplartine^23,52^, and nerolidol^40^. Furthermore, it is known that praziquantel also causes morphological changes in the tegument of schistosomes^42,53^. Studies on anthelmintic activity of other lignans were described by de Paula Carlis and colleagues^54^, in a study which evaluated the effects of the hinokinin, cubebin, and dihydrocubebin isolated from *Piper cubeta* fruits, against eggs and third stage larvae of sheep gastrointestinal nematodes (*Haemonchus contortus* and *Trichostrongylus* sp). The authors reported that lignans caused serious lesions on the surface of parasites that led to death. Unlike nematodes, which are protected by a cuticle, schistosomes are covered by a living syncytium, called the tegument. Data from the present study clearly show that *threo*-austrobailignan-6 and verrucosin led to a pronounced change in the tegument of *S. mansoni*. Morphological alterations may not always result in the death of a parasite^42,53,55^, but the tegumental damage observed in this study could be a mechanism through which neolignans kill the schistosomes. From the point of view of an in vivo study, in addition to the direct effect on the survival of the schistosomes, tegumental alterations might result in exposure of the worm antigens to the host’s immune system^56^. Furthermore, considering that differential appearance of morphological changes was noted, it will be interesting to see the effect when the two drugs are combined in future experiments.

Taken together, this finding indicates that both *threo*-austrobailignan-6 and verrucosin have a significant antischistosomal effect and the morphological alterations could be a mechanism through which these neolignans kill the helminths. However, the exact mechanism is unclear and needs further investigation. In *T. cruzi*, Brito and coworkers^21^ reported that dibenzylbutane neolignans act on the plasma membrane of the parasite and induce alterations in the mitochondrial membrane potential. Moreover, because the redox system is responsible for the survival of many parasites, the authors demonstrated that neolignans cause alterations in the ROS production. Studies have reported that some antischistosomal compounds, such as licochalcone A, elicit mitochondrial and cellular membrane alterations in *S. mansoni*^57^. In addition, a wide range of natural compounds are known to generate ROS in *S. mansoni*, and the induction of oxidative stress has been considered an attractive treatment strategy for schistosomiasis drug discovery^57–59^. Accordingly, the possibility of *threo*-austrobailignan-6 and verrucosin causing disturbance in both ROS and the activities of enzymes associated with maintaining redox balance cannot be excluded.

### *Threo*-austrobailignan-6 and verrucosin did not exhibit cytotoxicity

Isolated neolignans were incubated with two mammalian cell lines, namely the African green monkey kidney epithelial cells (Vero) and human epithelial cells (HaCaT) to evaluate their in vitro cytotoxic concentration 50% (CC_50_). The selectivity index (SI) was calculated as the ratio between the CC_50_ values for the human cells and the EC_50_ values for *S. mansoni*. As shown in Table 1, the tested compounds were non-cytotoxic to both animal and human cell lines. Accordingly, high SI values were determined, with SI > 17.8 and > 26.8 for *threo*-austrobailignan-6 and verrucosin, respectively. In line with this study, the low cytotoxic profile of neolignans from *S. cernuus* had been reported^21^. The SI of a compound is a widely accepted criterion used to express a drug’s in vitro efficacy^60,61^. Since the WHO establishes a SI value ≥ 10 for the selection of candidate anthelminthic compounds^49,62^, this study demonstrated that *threo*-austrobailignan-6 and verrucosin are highly selective antischistosomal agents.

In conclusion, neolignans *threo*-austrobailignan-6 and verrucosin, isolated from the freshwater plant *S. cernuus,* exhibited promising antiparasitic activity against blood fluke *S. mansoni*, inducing severe tegumental damage and a significant decrease in the production of eggs. In addition, these compounds displayed low cytotoxicity potential against both animal and human cell lines, resulting in relevant selectivity indices. Considering the promising chemical and biological properties of *threo*-austrobailignan-6 and verrucosin, these lignans could be used as starting points to develop new antischistosomal agents.

## Methods

### General procedures

^1^H and ^13^C NMR spectra were recorded on an Ultrashield 300 Bruker Avance III spectrometer (Billerica, MA, USA), operating, respectively, at 300 and 75 MHz. As a solvent and internal standard were used CDCl_3_ (Aldrich) and TMS (Aldrich). Chemical shifts are reported in δ units (ppm) and coupling constants (*J*) in Hz. ESI-HRMS spectra were measured on a Bruker Daltonics MicroTOF QII spectrometer. Silica gel 60 (Merck, 63–210 mesh) and Sephadex LH-20 (GE) were used for column chromatographic separation procedures whereas silica gel 60 PF_254_ (Merck) was used for analytical (0.25 mm) and preparative (1.00 mm) thin-layer chromatography (TLC).

### Plant material

The use of plant parts in the present study complies with the international, national, and institutional guidelines. *S. cernuus* leaves were obtained from a local producer of ornamental plants in the city of Suzano, São Paulo State, Brazil in August/2019 and, after collection, received a registration code A4123E4 at SISGEN (National System for the Management of Genetic Heritage and Associated Traditional Knowledge—Ministry of the Environment, Brazil). Botanical identification was performed by Dr. Fátima Otavina de Souza Buturi, from University of São Judas Tadeu, and Dr. Oriana Aparecida Fávero, from Mackenzie Presbyterian University. A voucher specimen (voucher number E. A. Ferreira—001) has been deposited at the SPF Herbarium of the Institute of Biosciences at the University of São Paulo.

### Isolation of *threo*-austrobailignan-6 and verrucosin

Dried leaves of *S. cernuus* (315 g) were powdered and exhaustively extracted using MeOH at room temperature to afford 91 g of crude extract after solvent evaporation at reduced pressure. This extract was resuspended in MeOH:H_2_O 1:1 and partitioned using hexane to afford 18.6 g of hexane phase. Part of this material (9.0 g) was subjected to column chromatography over silica gel eluted with *n*-hexane containing increasing amounts of EtOAc to afford fourteen fractions (A–N). After an initial screening, fractions C and G displayed activity against *S. mansoni* and were selected for further purification procedures. Therefore, fraction C (671 mg) was subjected to column chromatography over silica gel eluted with *n*-hexane containing increasing amounts of EtOAc to afford six fractions (C1–C6). Fraction C3 (232 mg) was chromatographed over a silica gel column eluted with *n*-hexane:EtOAc 3:2 to afford the fraction C3-4 (25 mg) which was purified by prep. TLC (20 × 20 cm^2^, toluene:Me_2_CO 7:3). This procedure gave *threo*-austrobailignan-6 (13 mg)^21^. Part of fraction G (760 mg) was chromatographed over Sephadex LH-20 eluted with *n*-hexane:CH_2_Cl_2_ 1:4 and CH_2_Cl_2_:acetone 3:2 to afford pure verrucosin (335 mg).

### NMR and ESI-HRMS data of *threo*-austrobailignan-6 and verrucosin

Threo*-austrobailignan-6*. White amorphous solid. ^1^H NMR (CDCl_3_), δ /ppm 6.80 (d, *J* = 7.9 Hz, H-5′), 6.70 (d, *J* = 7.8 Hz, H-5), 6.67 (d, *J* = 1.7 Hz, H-2), 6.60 (d, *J* = 1.7 Hz, H-2′), 6.57–6.53 (m, H-6/H-6′), 5.93 (s, OCH_2_O), 3.85 (s, OCH_3_), 2.57 (dd, *J* = 13.5 and 6.5 Hz, H-7b), 2.56 (dd, *J* = 13.5 and 6.5 Hz, H-7′b), 2.39 (dd, *J* = 13.5 and 8.0 Hz, H-7a), 2.33 (dd, *J* = 13.5 and 8.0 Hz, H-7′a), 1.76–1.67 (m, H-8/H-8′), 0.86 (d, *J* = 5.9 Hz, H-9), 0.81 (d, *J* = 6.6 Hz, H-9′). ^13^C NMR (CDCl_3_), δ /ppm 147.3 (C-3), 146.3 (C-3′), 145.4 (C-4), 143.5 (C-4′), 135.5 (C-1), 133.5 (C-1′), 121.8 (C-6), 121.6 (C-6′), 114.0 (C-5′), 111.3 (C-2′), 109.3 (C-2), 107.9 (C-5), 100.7 (OCH_2_O), 55.8 (OCH_3_), 41.1 (C-7), 41.0 (C-7′), 37.9 (C-8), 37.8 (C-8′), 13.9 (C-9), 13.8 (C-9′). ESI-HRMS *m/z* 327.1594 [M—H]^−^ (calcd for C_20_H_23_O_4_ 327.1596).

*Verrucosin*. White amorphous solid. ^1^H NMR (CDCl_3_), δ /ppm 6.97–6.76 (m, H-2/H-2′, H-5/H-5′, and H-6/H-6′), 5.03 (d, *J* = 8.7 Hz, H-7), 5.54 (4-OH), 5.49 (4′-OH),4.33 (d, *J* = 9.3 Hz, H-7′), 3.84 (s, 3-OMe), 3.78 (s, 3′-OMe), 2.20–2.11 (m, H-8), 1.75–1.64 (m, H-8′), 0.98 (d, *J* = 6.5 Hz, H-9), 0.59 (d, *J* = 7.0 Hz, H-9′). ^13^C NMR (CDCl_3_), δ /ppm 146.5 (C-3), 146.2 (C-3′), 145.2 (C-4), 144.6 (C-4′), 133.2 (C-1), 132.8 (C-1′), 119.9 (C-6′), 119.3 (C-6), 114.2 (C-5), 113.9 (C-5′), 109.7 (C-2′), 109.4 (C-2), 87.3 (C-7), 83.1 (C-7′), 55.9 (3-/3′-OMe), 47.8 (C-8), 46.0 (C-8′), 14.9 (C-9′), 14.5 (C-9). ESI-HRMS *m/z* 345.1798 [M + H]^+^ and 367.1518 [M + Na]^+^ (calcd for C_20_H_25_O_5_ 345.1702 and for C_20_H_24_O_5_Na 367.1521).

All the structural data collected are presented in the Supplementary Information (Fig. S1-S6).

### Drugs and reagents

Dulbecco’s Modified Eagle Medium (DMEM) modified to contain 4 mM of L-glutamine, 4500 mg/L glucose, and 1 mM sodium pyruvate, Roswell Park Memorial Institute (RPMI) 1640 medium, trypsin/EDTA solutions, heat-inactivated fetal calf serum, and penicillin G-streptomycin solutions (10,000 U/ml penicillin G sodium salt, 10 mg/ml streptomycin sulfate) were obtained from Vitrocell (Campinas, SP, Brazil). HEPES buffer, thiazolyl blue tetrazolium bromide (MTT), and dimethyl sulfoxide (DMSO) were purchased from Sigma (St. Louis, MO, USA). Praziquantel was kindly provided by Ecovet Indústria Veterinária Ltda (São Paulo, SP, Brazil). In all in vitro experiments, compounds were solubilized in DMSO.

### Animals, parasites, and cell lines

The life cycle of *S. mansoni* (BH strain) is maintained by routine passage through *Biomphalaria glabrata* snails and Swiss mice at Guarulhos University (UNG, Guarulhos, SP, Brazil). Rodents, three weeks-old, were purchased from Animais de Laboratório Criação e Comércio (Paulínea, SP, Brazil). Both mice and snails were kept under environmentally controlled conditions (25 °C; humidity of 50%), with free access to food and water. Cercariae of *S. mansoni* were obtained from infected intermediate host snails in our laboratories as described previously^63,64^.

HaCaT (human epithelial cells) and Vero (monkey kidney cells) were obtained from the Banco de Células do Rio de Janeiro (BCRJ, RJ, Brazil) and the American Type Culture Collection (ATCC CCL-81; Manassas, VA, USA), respectively. Cells were cultured in DMEM medium supplemented with 10% heat-inactivated fetal bovine serum and antibiotics (100 U/mL penicillin and 100 μg/mL streptomycin) at 37 °C in a humidified atmosphere containing 5% CO_2_. They were maintained in 25 cm^2^ culture flasks (Corning, Tewksbury, MA, USA) and harvested using 0.25% trypsin in 0.2 g/L EDTA solution.

### Antiparasitic assay

The antischistosomal assay was performed as previously described^65,66^. Briefly, *S. mansoni* adult parasites were obtained from infected mice at day 42 post-infection (parasite ex vivo). One adult male and one adult female worms (i.e. one worm pairs) were incubated in flat bottom 24-well plates (Corning, New York, NY, USA) in the presence of *threo*-austrobailignan-6 and verrucosin (started at 50 µM and followed a twofold dilution series) in RPMI 1640 culture medium supplemented with 5% inactivated fetal calf serum and containing antibiotics (100 U/mL penicillin and 100 μg/mL streptomycin). The control schistosomes were assayed in a culture medium and 0.5% DMSO (representing the highest concentration of solvent). Each concentration (50, 25, 12.5, 6.25, 3.12 µM) was tested in five replicates, and the experiments were repeated three times. Parasites were kept for 72 h (37 °C, 5% CO_2_), and their viability was monitored microscopically at 2, 24, 48, and 72 h using both a Motic AE2000 inverted microscope (Vancouver, Canada) and a Leica EZ4E stereomicroscope (Wetzlar, Germany). The death of adult schistosomes was defined as no movement observed for at least 1 to 2 min of examination, whereas parasites with any body movement were considered viable^67^. The percentage of viable adult schistosomes was calculated considering worms exposed to compounds *vs* control worms. For assessment of the reproductive fitness of *S. mansoni*, the number of eggs was counted daily using an inverted microscope (Motic) as previously described^68^.

### Scanning electron microscopy investigation

Scanning electron microscopy studies were performed as previously described^69,70^. Briefly, adult worms (treated and control groups) were fixed in 2.5% glutaraldehyde, and mounted specimens were coated with gold sputter (Denton Vacuum LLC, Moorestown, NJ, USA) and photographed using a high-resolution scanning electron microscope (Jeol-JSM-6460LV, Tokyo, Japan).

### Cytotoxicity assay

The MTT assay was used to evaluate the cytotoxic activity as previously described^71,72^. Briefly, cells were seeded (2 × 10^3^/well in 96-well culture plates) and incubated with *threo*-austrobailignan-6 and verrucosin (started at 500 µM and followed a threefold dilution series) for 72 h at 37 °C and 5% CO_2_. MTT solution was added to each well and the absorbance was read on an Epoch Microplate Spectrophotometer (BioTek Instruments, Winooski, VT, USA) at 595 nm. The selectivity indices (SI) were calculated by dividing the 50% cytotoxic concentration (CC_50_) obtained on cells with 50% effective concentration (EC_50_) values determined on schistosomes^73^.

### Statistical analysis

Statistical analyses were performed using Graph Pad Prism software 8.0 (San Diego, CA, USA). Data are presented as the mean ± standard deviation (SD) of at least three independent experiments, EC_50_ and, CC_50_ values were calculated using sigmoid dose–response curves^74^. For *S. mansoni* egg production, significant differences were determined by one-way analysis of variance (ANOVA) and applying Tukey's test for multiple comparisons with a level of significance set at *P* < 0.05.

### Ethical approval

Animal studies are reported in compliance with the ARRIVE guidelines. The protocol for experimental design was reviewed and approved by the Committee for the Ethical Use of Animals in Experimentation of the Guarulhos University (Guarulhos, SP, Brazil; protocol ID 47/20) in conformity with the Brazilian law for Guidelines for Care and Use of Laboratory Animals.

## Supplementary Information

Supplementary Information.

## Data Availability

The raw data that support the findings of this study are available from the corresponding author upon reasonable request.
