# Supplementary material for: Neolignans isolated from Saururus cernuus L. (Saururaceae) exhibit efficacy against Schistosoma mansoni
Source: Sci Rep. 2022 Nov 11;12:19320. doi: 10.1038/s41598-022-23110-2 (PMC9652300; doi:10.1038/s41598-022-23110-2)
Supplement: Supplementary file 1 — Supplementary Information. [file 41598_2022_23110_MOESM1_ESM.pdf]

Threo-austrobailignan-6 and verrucosin, two neolignans isolated from *Saururus cernuus* L. (Saururaceae), exhibit efficacy against *Schistosoma mansoni*

Juliana R. Brito[1], Daniel B. Roquini[2], Beatriz C. Parra[2], Marina M. Gonçalves[3], Dalete Christine S. Souza[3], Edgard A. Ferreira[4], Maria C. Salvadori[5], Fernanda S. Teixeira[5], Polrat Wilairatana[6,*], João Henrique G. Lago[3,*], Josué de Moraes[2,*]

[1]Institute of Environmental, Chemical and Pharmaceutical Sciences, Federal University of São Paulo, Diadema, SP, 09972-270, Brazil
[2]Research Center on Neglected Diseases, Guarulhos University, Guarulhos, SP, 07023-070, Brazil
[3]Center for Natural and Human Sciences, Federal University of ABC, Santo André, SP, 09210-180, Brazil
[4]School of Engineering, Mackenzie Presbyterian University, São Paulo, SP, 01302-907, Brazil
[5]Institute of Physics, University of São Paulo, São Paulo, SP, 05508-090, Brazil
[6]Department of Clinical Tropical Medicine, Faculty of Tropical Medicine, Mahidol University, Bangkok 10400, Thailand

[*]Corresponding authors: joao.lago@ufabc.edu.br (J.H.G.L.); polrat.wil@mahidol.ac.th (P.W.); moraesnpdn@gmail.com (J.d.M.).

Supplementary Information

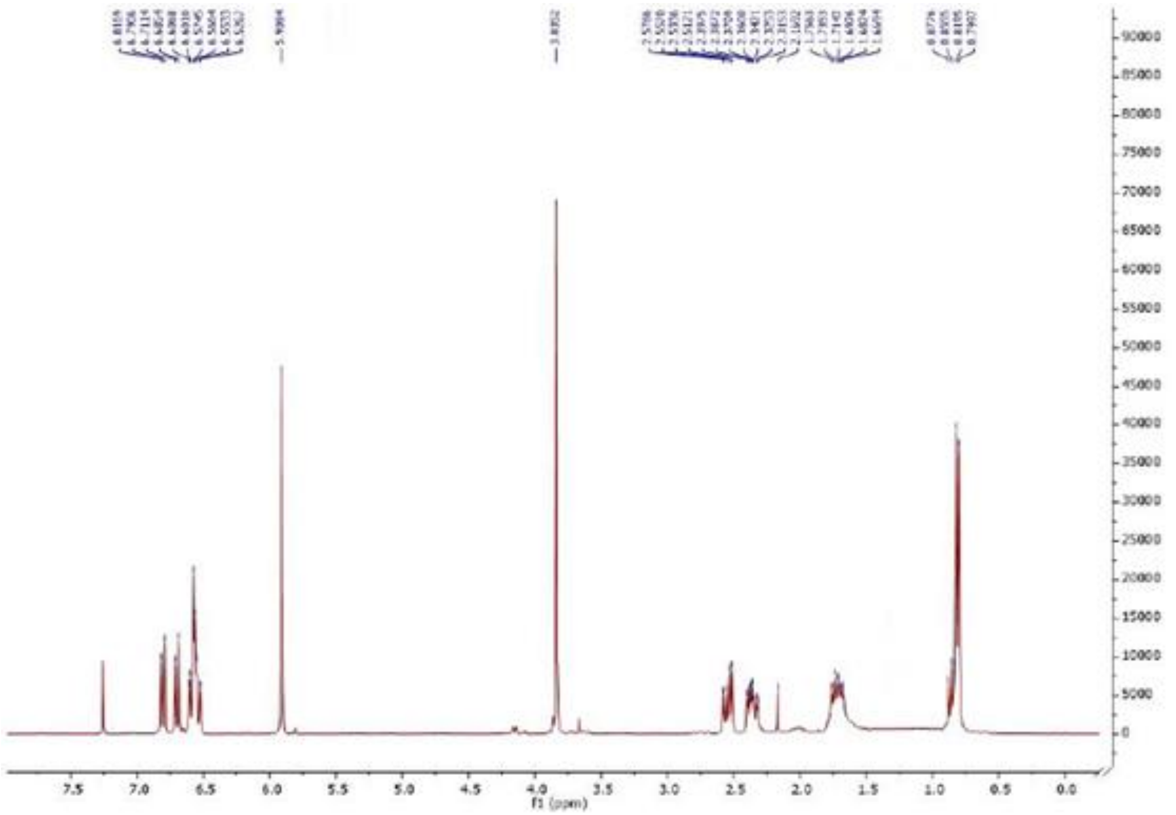

Figure S1. ^{1}H NMR spectrum of *threo*-austrobailignan-6 (δ, $CDCl_3$, 300 MHz).

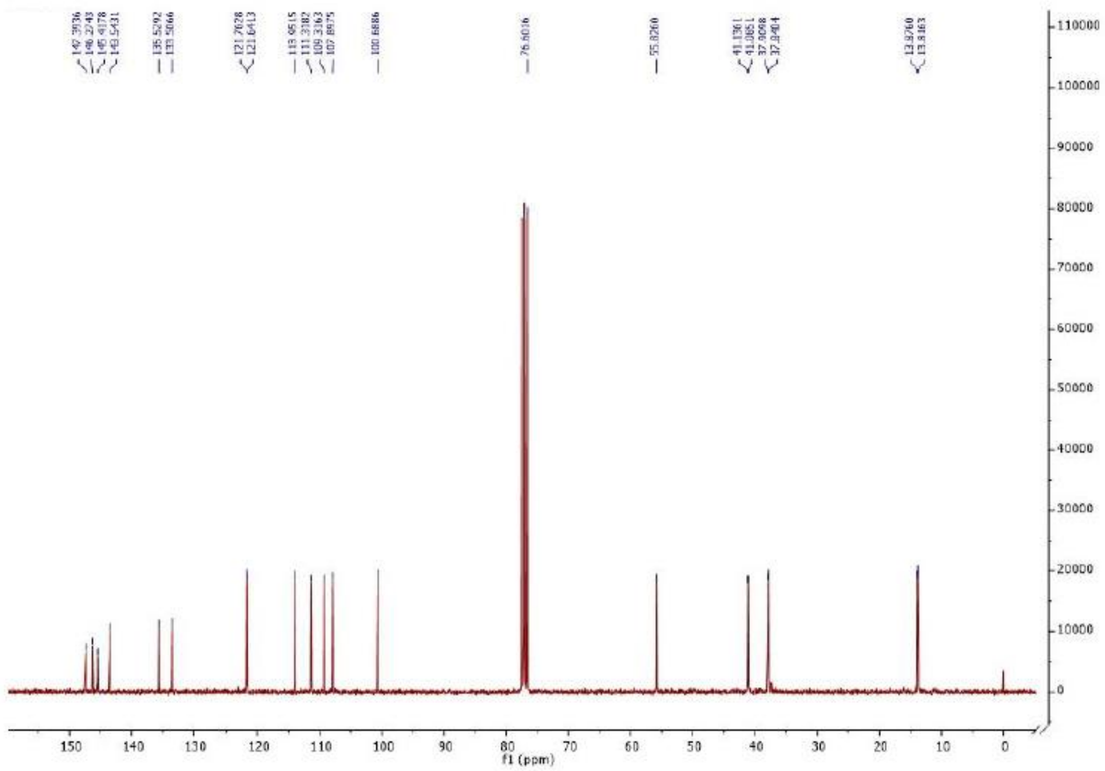

Figure S2. ^{13}C NMR spectrum of *threo*-austrobailignan-6 (δ, $CDCl_3$, 75 MHz).

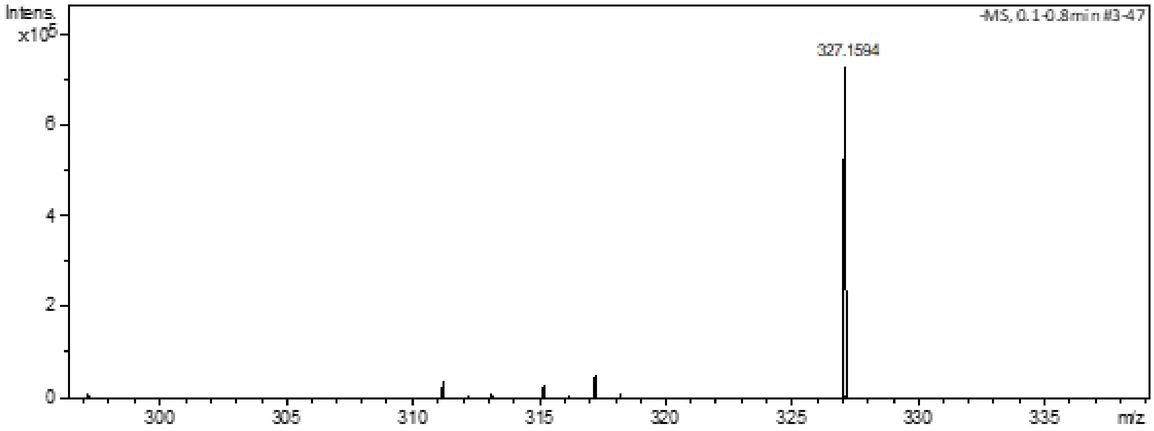

Figure S3. ESI-HRMS spectrum of *threo*-austrobailignan-6 (negative mode).

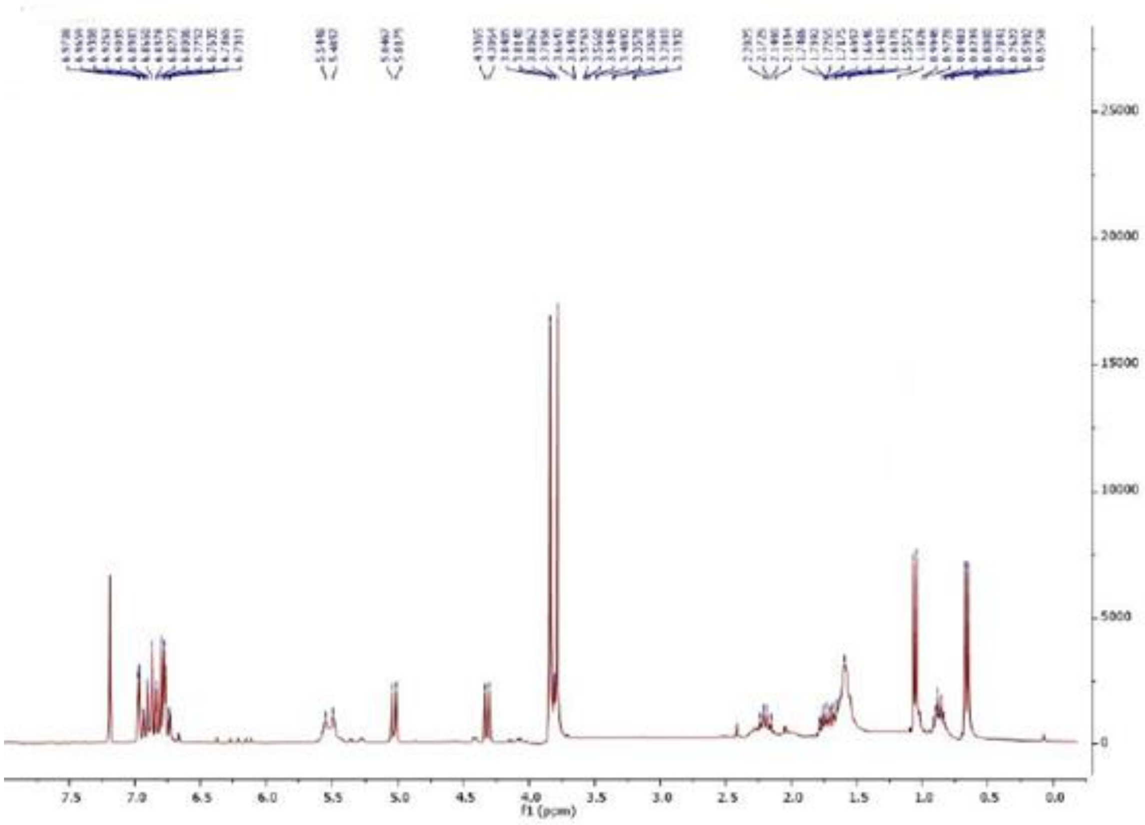

Figure S4. ^{1}H NMR spectrum of verrucosin (δ, $CDCl_3$, 300 MHz)

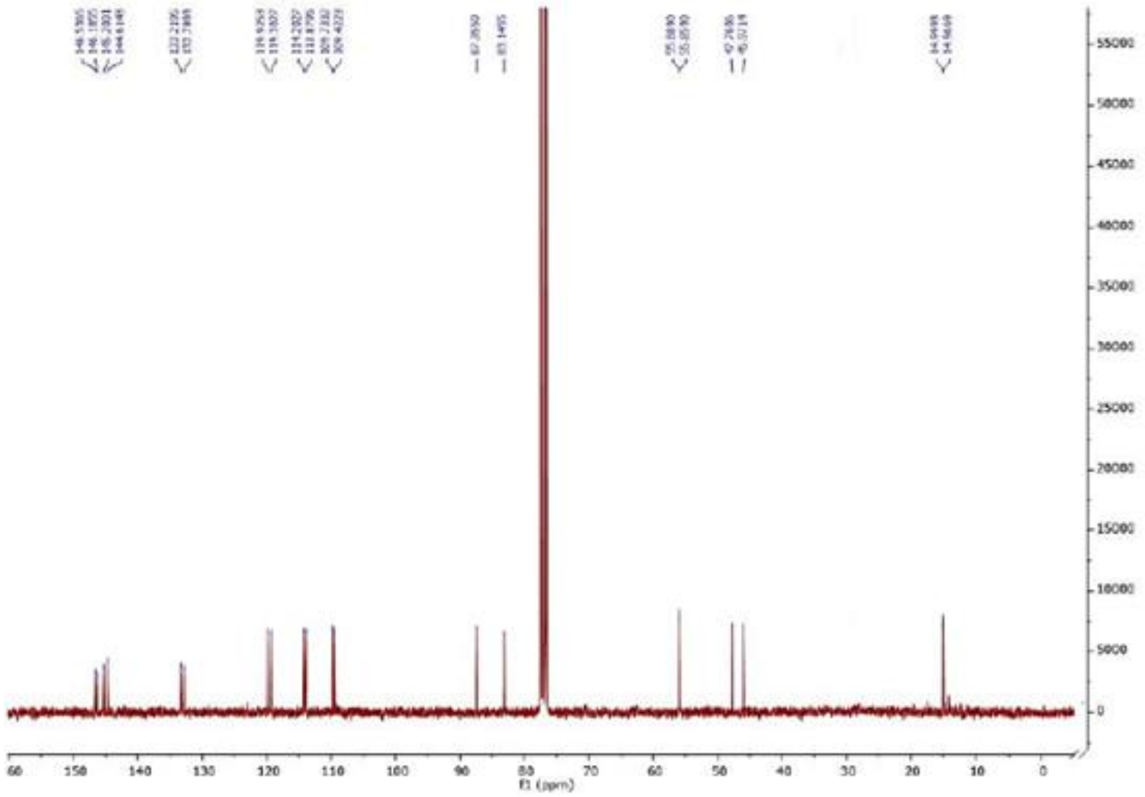

Figure S5. ^{13}C NMR spectrum of verrucosin (δ, $CDCl_3$, 75 MHz)

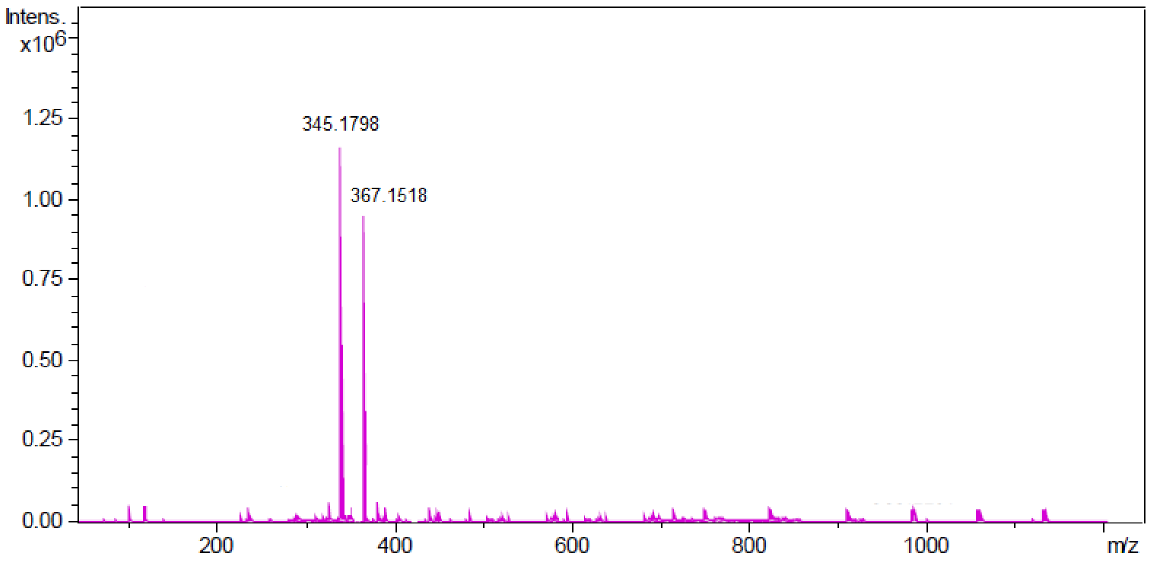

Figure S6. ESI-HRMS spectrum of verrucosin (positive mode).
